# Future perspective and clinical applicability of the combined use of plasma phosphorylated tau 181 and neurofilament light chain in Subjective Cognitive Decline and Mild Cognitive Impairment

**DOI:** 10.1038/s41598-024-61655-6

**Published:** 2024-05-17

**Authors:** Giulia Giacomucci, Salvatore Mazzeo, Assunta Ingannato, Chiara Crucitti, Silvia Bagnoli, Sonia Padiglioni, Lucrezia Romano, Giulia Galdo, Filippo Emiliani, Daniele Frigerio, Camilla Ferrari, Valentina Moschini, Carmen Morinelli, Antonella Notarelli, Sandro Sorbi, Benedetta Nacmias, Valentina Bessi

**Affiliations:** 1 Department of Neuroscience, Psychology, Drug Research and Child Health, University of Florence, Azienda Ospedaliero-Universitaria Careggi, Largo Brambilla, 3, 50134 Florence, Italy; 2 Regional Referral Centre for Relational Criticalities - Tuscany Region, University of Florence, Florence, Italy; 3 Research and Innovation Centre for Dementia-CRIDEM, AOU Careggi, Florence, Italy; 4 University of Florence, Florence, Italy; 5 SOD Neurologia I, Dipartimento Neuromuscolo-Scheletrico e degli Organi di Senso, AOU Careggi, Florence, Italy; 6 IRCCS Fondazione Don Carlo Gnocchi, Florence, Italy; 7 Present Address: Vita-Salute San Raffaele University, Milan, Italy; 8 Present Address: IRCCS Policlinico San Donato, San Donato Milanese, Italy

**Keywords:** Plasma biomarkers, p-tau181, NfL, Subjective Cognitive Decline, Mild Cognitive Impairment, Alzheimer’s Disease, Neurology, Dementia

## Abstract

We aimed to assess diagnostic accuracy of plasma p-tau181 and NfL separately and in combination in discriminating Subjective Cognitive Decline (SCD) and Mild Cognitive Impairment (MCI) patients carrying Alzheimer’s Disease (AD) pathology from non-carriers; to propose a flowchart for the interpretation of the results of plasma p-tau181 and NfL. We included 43 SCD, 41 MCI and 21 AD-demented (AD-d) patients, who underwent plasma p-tau181 and NfL analysis. Twenty-eight SCD, 41 MCI and 21 AD-d patients underwent CSF biomarkers analysis (Aβ1-42, Aβ1-42/1–40, p-tau, t-tau) and were classified as carriers of AD pathology (AP+) it they were A+/T+ , or non-carriers (AP−) when they were A−, A+/T−/N−, or A+/T−/N+ according to the A/T(N) system. Plasma p-tau181 and NfL separately showed a good accuracy (AUC = 0.88), while the combined model (NfL + p-tau181) showed an excellent accuracy (AUC = 0.92) in discriminating AP+ from AP− patients. Plasma p-tau181 and NfL results were moderately concordant (Coehn’s k = 0.50, *p* < 0.001). Based on a logistic regression model, we estimated the risk of AD pathology considering the two biomarkers: 10.91% if both p-tau181 and NfL were negative; 41.10 and 76.49% if only one biomarker was positive (respectively p-tau18 and NfL); 94.88% if both p-tau181 and NfL were positive. Considering the moderate concordance and the risk of presenting an underlying AD pathology according to the positivity of plasma p-tau181 and NfL, we proposed a flow chart to guide the combined use of plasma p-tau181 and NfL and the interpretation of biomarker results to detect AD pathology.

## Introduction

In recent years the definition of Alzheimer’s Disease (AD) underwent a major change, shifting from a pure clinical construct to a clinical-biological entity^1,2^. The new clinical-biological definition of AD is based on the in vivo demonstration of typical neuropathologic changes (i.e. deposition of β-amyloid plaques and neurofibrillary tangles of hyperphosphorylated tau) that could be detected many years before the beginning of clinical manifestations^1^. Currently used biomarkers (CSF biomarkers^3^, PET neuroimaging and brain MRI^4,5^) are highly accurate in detecting AD pathology in the early stage, such as in patients with Subjective Cognitive Decline (SCD) and Mild Cognitive Impairment (MCI)^6,7^. However, their use on large populations is extremely limited by their cost, insufficient accessibility, or invasiveness. For these reasons, the search for biomarkers has progressively shifted towards a more accessible substrate, such as peripheral blood. Blood-based biomarkers are promising tools for the early detection of AD which might be used also at the primary care levels^8^. Among these biomarkers, plasma p-tau181 is highly accurate in discriminating AD from other neurodegenerative disease and from healthy controls, even in prodromal stages^9,10^. Moreover, it showed a high accuracy in detecting AD pathology in SCD^11^. Regarding NfL, recent studies have shown that this biomarker might be useful to predict progression of cognitive decline in SCD and MCI^12^.

At the present time, plasma biomarkers are not regularly used in clinical practice, and they still remain restricted to research settings. Current research is also aiming to explore combinations of plasma biomarkers in order to reach the highest accuracy in discriminating AD from non-AD neurodegenerative diseases and in detecting AD pathology. However, despite the urgent need to focus of preclinical and prodromal stages of AD, to the best of our knowledge, only few studies were conducted specifically on SCD patients trying to explore the role of combined biomarkers^13,14^. Moreover, there are no indication about how to interpret the results of plasma biomarkers when used in combination.

In this perspective, we hypothesized that the combined use of plasma p-tau181 and NfL may be more accurate than single biomarkers in identifying those patients with an underlying AD pathology. Therefore, we aimed to assess the diagnostic accuracy of plasma p-tau181 and NfL alone, and also of a combined model which included both biomarkers (NfL + p-tau181) in discriminating SCD and MCI patients carrying AD pathology from non-carriers; moreover, we investigated the concordance between the results of plasma p-tau181 and NfL. Finally, we proposed a flow chart for the interpretation of plasma p-tau181 and NfL in early stages of cognitive decline and the potential future clinical applicability.

## Materials and methods

### Participants

Between July 2018 and September 2023, we consecutively enrolled 105 white Italian patients (43 SCD, 41 MCI and 21 AD demented) referred to the centre for Alzheimer’s Disease and Adult Cognitive Disorders of Careggi Hospital in Florence.

Patients met the following inclusion criteria:

- Receiving a clinical diagnosis of AD dementia according to the NIA-AA criteria, including the atypical variant^15^.
- Receiving a clinical diagnosis of MCI according to NIA-AA criteria^16^.
- Receiving a clinical diagnosis of SCD according to SCD-I criteria^17^.

Exclusion criteria were: history of head injury, current neurological and/or systemic disease, symptoms of psychosis, major depression, substance use disorder.

At baseline, patients underwent comprehensive family and clinical history, neurological examination and extensive neuropsychological investigation (described in detail elsewhere^18^), blood collection for measurement of plasma NfL and p-tau181 concentration and genetic analysis.

We defined age at baseline as the age at the time of plasma collection, disease duration as timeframe of onset of symptoms relative to baseline examination, positive family history of dementia as having one or more first-degree relatives with documented cognitive decline.

Renal function was categorized as either impaired or not impaired based on estimated glomerular filtration rate (eGFR; considered impaired if < 60 mL/min/1.73 m^2^). eGFR was recorded only in patients with impaired renal function.

*APOE* genotyping was available for 103 patients (41 SCD, 41 MCI, 21 AD-d). A total of 90 patients (28 SCD, 41 MCI, 21 AD-d) underwent CSF collection for Aβ1-42, Aβ1-42/Aβ1-40, t-tau and p-tau. Normal values for CSF biomarkers were: Aβ1-42 > 670 pg/mL, Aβ1-42/Aβ1-40 > 0.062, t-tau < 400 pg/mL and p-tau < 60 pg/mL^19^.

Plasma p-tau181 and NfL were dichotomized considering the cut-off previously identified for discriminating AP+ from AP− patients in SCD and MCI,: for plasma p-tau181 positive if ≥ 2.69 pg/mL, negative if < 2.69 pg/mL; for plasma NfL, negative if < 19.45 pg/mL in SCD and < 20.49 pg/mL in MCI, positive if ≥ 19.45 pg/mL in SCD and ≥ 20.49 pg/mL in MCI^11,12^.

Thirty-six patients (25 SCD, 5 MCI and 6 AD-d) underwent amyloid-PET. Twenty-eight patients (17 SCD, 5 MCI and 6 AD-d) underwent both CSF analysis and amyloid-PET scans. Ninety-five patients (37 SCD, 39 MCI and 19 AD-d) also underwent fluorodeoxyglucose positron emission tomography (FDG-PET). Methods used for blood and CSF collection, *APOE* genotyping, CSF analysis, brain FDG-PET and amyloid-PET acquisition and rating are described in further detail elsewhere^11,12,20,21^.

Study procedures and data analysis were performed in accordance with the Declaration of Helsinki and with the ethical standards of the Committee on Human Experimentation of our Institute. The study was approved by the local Institutional Review Board (Comitato Etico Regione Toscana—Area Vasta Centro) (reference 15691oss). All individuals involved in this research agreed to participate and agreed to have details and results of the research about them published.

### Classification of patients according to ATN system

Based on biomarker results, patients were classified according to the NIA-AA Research Framework (amyloid/tau/neurodegeneration A/T/N system)^1^. Patients were rated as A+ if at least one of the amyloid biomarkers (CSF or amyloid PET) revealed the presence of Aβ pathology, and as A− if none of the biomarkers revealed the presence of Aβ pathology. In the case of discordant CSF and amyloid PET results, we considered only the pathological result. Patients were classified as T+ or T− if CSF p-tau concentrations were higher or lower than the cut-off value, respectively. Patients were classified as N+ if at least one neurodegeneration biomarker was positive (CSF t-tau higher than the cut-off value or positive FDG-PET). Patients were further classified as carrier of AD pathology (AP+) when A+ was associated with T+ (regardless of N classification), or non-carriers (AP−) when they were classified as A− (regardless of T and N classification), or A+/T−/N−, or A+/T−/N+^11^. Using a previously described procedures, patients were furtherly classified according both to diagnosis (SCD, MCI, AD-d) and ATN classification (AP− and AP+) as follows: SCD AP−, SCD AP+ , MCI AP−, MCI AP+ , AD-d (all the AD-d patients were AP+)^11^.

### Plasma p-tau181 and NfL analysis

Blood was collected by venipuncture into standard polypropylene EDTA test tubes (Sarstedt, Nümbrecht, Germany) and centrifuged within 2 h at 1300 rcf at 4 °C for 10 min. Plasma was isolated and stored at − 80 °C until testing. Plasma NfL analysis was performed with Simoa NF-Light SR-X kit (cat. No. 103400) for human samples provided by Quanterix Corporation (Lexington, Massachusetts) on the automatized Simoa SR-X platform (GBIO, Hangzhou, China), following the manufacturer’s instructions. The lower limits of quantification and detection provided by the kit were 0.316 and 0.0552 pg/mL, respectively. The plasma NfL concentrations in all samples were detected in a single run. Quality controls with a low NfL concentration of 5.08 pg/mL and a high NfL concentration of 169 pg/mL were included in the array and assessed with samples. The NfL assay results are consistent with the expected values, exhibiting a coefficient of variation below 20%.

The Simoa Human p-tau181 Advantage V2 kit (item #103714, provided by Quanterix Corp.—Billerica, MA, USA) was used for the quantitative determination of p-tau181 in plasma sample. The kit analytical lower limit of quantification (LLOQ) value was 0.085 pg/mL, instead the kit limit of detection (LOD) was 0.041 pg/mL (range 0.018–0.060 pg/mL). For the run setup, 7 calibrators and 2 controls, provided by Quanterix, were required for the analysis. Calibrators were used to set a calibration curve of serially measurements, controls were the lower and higher target concentration. Plasma samples and controls were diluted 4×. Calibrators, controls and samples were run in duplicate, detected in a single run basis^11,12,22^.

### Statistical analysis

All statistical analyses were performed using IBM SPSS Statistics software version 25 (SPSS Inc., Chicago, Illinois) and the computing environment R4.2.3 (R Foundation for Statistical Computing, Vienna, 2013). All *p* values were two-tailed and the significance level for all analyses was set at *p* = 0.05. Distributions of all variables were assessed using the Shapiro–Wilk test. As both plasma p-tau181 and NfL were not normally distributed, we applied log_10_ transformation. This transformation resulted in a more normally distributed dataset that met the assumptions of the statistical tests that we planned to use. We conducted descriptive statistics using means and standard deviation for continuous variables and frequencies or percentages and 95% confidence intervals (CIs) for categorical variables. We used the t-test for comparison between two groups, one-way analysis of variance (ANOVA) with Bonferroni post hoc test for comparisons among three or more groups, Pearson’s correlation coefficient to evaluate correlations between groups’ numeric measures, and chi- squared tests to compare categorical data. To adjust for possible confounding factors, we used multiple regression analysis. We performed a logistic regression analysis to define a combined model including plasma p-tau181 and NfL. We constructed receiver-operating characteristic (ROC) curves to evaluate the performance of plasma p-tau181, NfL and the combined model (NfL + p-tau in predicting ATN status. We used binomial logistic regression to ascertain the effect of plasma p-tau181 and NfL on the risk of presenting AD pathology. We calculated the size effect using Cohen’s d for normally distributed numeric measures, η^2^ for ANOVA and Cramer’s V for categorical data. Cohen’s k was used to explore concordance between plasma p-tau181 and NfL.

### Ethics approval and consent to participate

Local ethics committees (Comitato Etico Regione Toscana—Area Vasta Centro) approved the study at each site, and all participants provided written informed consent. The study was conducted according to the Declaration of Helsinki.

## Results

### Distribution of plasma p-tau181 and NfL across diagnostic groups

Demographic features and differences among diagnostic groups are summarized in Table 1. An MCI patient and two AD-d patients had impaired renal function (eGFR 47.7 mL/min/1.73 m^2^, 58.3 mL/min/1.73 m^2^, 53.0 mL/min/1.73 m^2^), with no differences in terms of proportion of renal impairment among the SCD, MCI and AD-d groups.

**Table 1:** Demographic features of Subjective Cognitive Decline (SCD), Mild Cognitive Impairment (MCI) and Alzheimer’s Disease dementia (AD-d) groups.

	SCD	MCI	AD-d
	N° 43	N° 41	N° 21
Age at onset in years	57.50 (± 9.30)*°	63.59 (± 10.68)*	**65.75 (± 6.32)°**
Age at plasma collection	67.15 (± 8.18)	70.08 (± 8.15)	70.04 (± 4.40)
Disease duration	**7.44 (± 7.00)**^**§**^	5.03 (± 5.82)	**2.57 (± 4.29)**^**§**^
Family history of AD	72.09%	60.97%	52.38%
Sex (M–F)	11–32	15–26	10–11
Years of education	12.60 (± 3.98)	12.30 (± 4.42)	11.08 (± 6.14)
MMSE	**27.69 (± 1.84)**^**#ç**^	**25.93 (± 2.11)**^**#**^**^**	**20.39 (± 5.11)**^**ç**^**^**
*APOE* ɛ4+	**31.70%**^**€**^	41.46%	**61.90%**^**€**^
Impaired renal function	0	1 (2.43%)	2 (9.52%)
Log p-tau181 (pg/ml)	**0.28 (± 0.20)**^**$**^	**0.34 (± 0.17)**^**£**^	**0.58 (± 0.17)**^**$£**^
Log NfL (pg/ml)	**1.13 (± 0.18)**^**&**^	1.18 (± 0.21)	**1.28 (± 0.12)**^**&**^

Values are reported as mean and standard deviation or frequencies or percentages for continuous variables and categorical variables respectively. Statistically significantly different values between the groups are reported as bold.

*M* males, *F* females, *MMSE* mini mental state examination.

Statistically significance: *p* < 0.05. *p = 0.005, Cohen’s d = 0.608; °*p* = 0.014, Cohen’s d = 1.038; ^§^*p* = 0.01, Cohen’s d = 0.839; ^#^*p* = 0.019, Cohen’s d = 0.889; ^ç^*p* < 0.001, Cohen’s d = 1.901; ^*p* < 0.001, Cohen’s d = 1.417; ^€^χ^2^ 5.20, *p* = 0.031, Cramer’s V 0.290; ^$^*p* < 0.001, Cohen’s d = 1.616; ^£^*p* = 0.001, Cohen’s d = 1.412; ^&^*p* = 0.004, Cohen’s d = 0.981.

Plasma p-tau181 levels were higher in AD-d as compared to MCI and to SCD patients (F = 13.72, *p* < 0.001, η^2^ = 0.212). NfL concentration was higher in AD-d than in SCD subgroup (F = 5.67, *p* = 0.005, η^2^ = 0.099). Plasma p-tau181 concentration was correlated with age at plasma collection (Pearson 0.262, *p* = 0.007) and age at onset (Pearson 0.306, *p* = 0.002). Similarly, NfL concentration was correlated with age at plasma collection (Pearson 0.544,* p* < 0.001) and age at onset (Pearson 0.461, *p* < 0.001). Plasma p-tau181 levels were higher in *APOE* ɛ4 carriers than in non-carriers (0.47 ± 0.17 vs 0.30 ± 0.27, *p* = 0.001). No differences in plasma NfL and p-tau181 levels were detected between males and females. The difference on plasma p-tau181 (between SCD and AD-d and between MCI and AD-d) and NfL levels (between SCD and AD-d) was confirmed after controlling for age, MMSE, *APOE* genotyping (p-tau181: F [4, 86] = 8.65, *p* < 0.001; NfL: F [4, 86]  = 11.97, *p* < 0.001).

### Distribution of plasma p-tau181 and NfL across diagnostic and biomarkers groups

The groups consisted of 20 SCD AP−, 8 SCD AP+ , 24 MCI AP−, 17 MCI AP+ , and 21 AD-d patients. Demographic variables are described in Table 2.

**Table 2:** Demographic features of diagnostic and biomarkers groups.

	SCD AP−	SCD AP+	MCI AP−	MCI AP+	AD-d
	N° 20	N° 8	N° 24	N° 17	N° 21
Age at onset in years	55.60 (± 9.69)*°	62.50 (± 10.9 9)	**60.13 (± 11.45)^**	70.31 (± 5.26)*^	**64.76 (± 5.38)°**
Age at plasma collection	**62.83 (± 8.33)**^**+#**^	**73.63 (± 6.05)**^**+**^	67.92 (± 8.71)	74.06(± 5.54)	**69.26 (± 5.45)**^**#**^
Disease duration	5.15 (± 6.18)	**10.25 (± 6.34)**^**&@**^	5.65 (± 6.77)	**3.33 (± 3.16)**^**&**^	**2.57 (± 3.46)**^**@**^
Family history of AD	**85.00%**^**§**^	75.00%	58.33%	64.70%	**52.38%**^**§**^
Sex (M–F)	4–16	4–4	8–16	7–10	10–11
Years of education	13.05 (± 3.32)	12.63 (± 5.09)	11.46 (± 4.55)	13.88 (± 3.87)	10.47 (± 5.34)
MMSE	**28.03 (± 1.56)**^**ç**^	**27.63 (± 2.10)**^**£**^	**26.18 (± 2.03)**^**$**^	**25.48 (± 2.18)**^**€**^	**20.70 (± 5.02)**^**ç,£,$,€**^
*APOE* ɛ4+	**35.00%**^**α**^	37.50%	**16.66%**^**β,γ**^	**76.47%**^**β**^	**61.90%**^**α,γ**^
Impaired renal function	0	0	1 (4.16%)	0	0
Log p-tau181 (pg/ml)	**0.23 (± 0.16)**^**a,b,c**^	**0.44 (± 0.08)**^**a,d**^	**0.22 (± 0.26)**^**d,e,f**^	**0.57 (± 0.13)**^**b,e**^	**0.59 (± 0.14)**^**c,f**^
Log NfL (pg/ml)	**1.07 (± 0.12)**^**g,h,i**^	**1.33 (± 0.18)**^**g,l**^	**1.11 (± 0.17)**^**l,m,n**^	**1.32 (± 0.20)**^**h,m**^	**1.29 (± 0.12)**^**i,n**^

Values are reported as mean and standard deviation or frequencies or percentages for continuous variables and categorical variables respectively. Statistically significantly different values between the groups are reported as bold.

*M* males, *F* females, *MMSE* mini mental state examination.

Statistically significance: *p* < 0.05, *p < 0.001, Cohen’s d = 1.887; °*p* = 0.014, Cohen’s d = 1.043; ^*p* = 0.006, Cohen’s d = 1.143; ^+^*p* = 0.006, Cohen’s d = 1.484; ^#^*p* < 0.001, Cohen’s d = 0.913; ^&^*p* = 0.037, Cohen’s d = 1.381; ^@^*p* = 0.010, Cohen’s d = 1.504; ^§^χ^2^ 5.03, *p* = 0.043, Cramer’s V 0.350; ^ç^*p* < 0.001, Cohen’s d = 1.974; ^£^*p* < 0.001, Cohen’s d = 1.801; ^$^*p* < 0.001, Cohen’s d = 1.431; ^€^*p* < 0.001, Cohen’s d = 1.235; ^α^χ^2^ 6.36, *p* = 0.020, Cramer’s V 0.269; ^β^χ^2^ 14.66, *p* < 0.001, Cramer’s V 0.598; ^γ^χ^2^ 9.75, *p* = 0.002, Cramer’s V 0.465; ^a^*p* = 0.002, Cohen’s d = 1.629; ^b^*p* < 0.001, Cohen’s d = 2.332; ^c^*p* < 0.001, Cohen’s d = 2.395; ^d^*p* = 0.048, Cohen’s d = 1.144; ^e^*p* < 0.001, Cohen’s d = 1.703; ^f^*p* < 0.001, Cohen’s d = 1.772; ^g^*p* = 0.003, Cohen’s d = 1.700; ^h^*p* < 0.001, Cohen’s d = 1.516; ^i^*p* = 0.001, Cohen’s d = 1.167; ^l^*p* = 0.014, Cohen’s d = 1.257; ^m^*p* = 0.001, Cohen’s d = 1.131; ^n^*p* = 0.004, Cohen’s d = 1.223.

Plasma p-tau181 levels were different among the groups also after adjusting for age, MMSE and *APOE* genotype (F [4, 73] = 18.29.16, *p* < 0.001). Post hoc analysis showed that p-tau181 concentration was higher in AD-d than in MCI AP− (*p* < 0.001, d = 1.772) and SCD AP− (*p* < 0.001, d = 2.395). No differences in plasma p-tau181 levels were found among AD-d, MCI AP + and SCD AP+ . P-tau181 concentration was higher in both MCI AP+ and SCD AP+ than in MCI AP− (MCI AP+ vs MCI AP− *p* < 0.001, d = 1.703; SCD AP+ vs MCI AP− *p* = 0.048, d = 1.144) and than in SCD AP− (MCI AP+ vs SCD AP− *p* < 0.001, d = 2.332; SCD AP+ vs SCD AP− *p* = 0.002, d = 1.629). No differences were detected between SCD AP− and MCI AP− (*p* = 1.00, d = 0.046).

**Figure 1:**
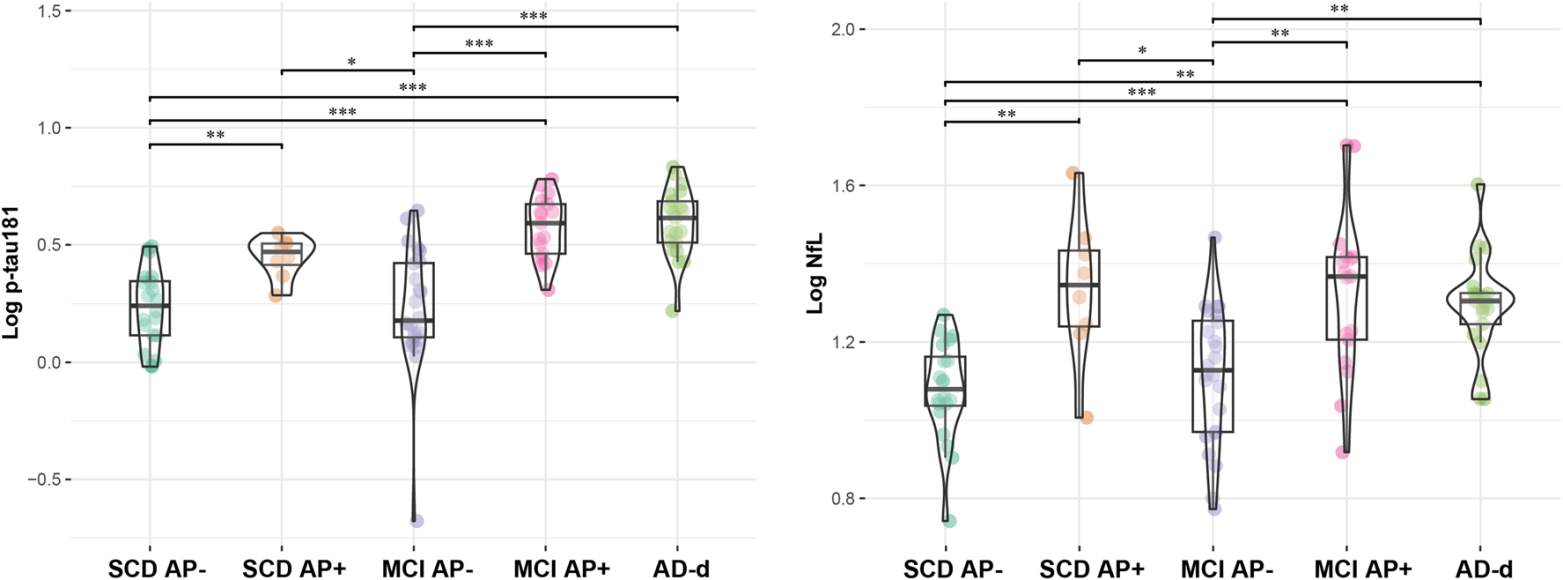
Plasma biomarkers levels across diagnosis/ATN groups. (**a**) Log p-tau181 levels across diagnosis/ATN groups. Values quoted in the y-axis indicate Log p-tau181 levels. Horizontal bars indicate significant differences between groups. SCD AP− vs SCD AP + *p* = 0.002, Cohen’s d = 1.629; SCD AP− vs MCI AP + *p* < 0.001, Cohen’s d = 2.332; SCD AP− vs AD-d *p* < 0.001, Cohen’s d = 2.395; SCD AP + vs MCI AP− *p* = 0.048, Cohen’s d = 1.144; MCI AP− vs MCI AP + *p* < 0.001, Cohen’s d = 1.703; MCI AP− vs AD-d *p* < 0.001, Cohen’s d = 1.772. (**b**) Log NfL levels across ATN biomarkers’ profile groups. Values quoted in the y-axis indicate Log NfL levels. Horizontal bars indicate significant differences between groups. SCD AP− vs SCD AP + *p* = 0.003, Cohen’s d = 1.700; SCD AP− vs MCI AP + *p* < 0.001, Cohen’s d = 1.516; SCD AP− vs AD-d *p* = 0.001, Cohen’s d = 1.167; SCD AP + vs MCI AP− *p* = 0.014, Cohen’s d = 1.257; MCI AP− vs MCI AP + *p* = 0.001, Cohen’s d = 1.131; MCI AP− vs AD-d *p* = 0.004, Cohen’s d = 1.223. *p < 0.05, **p < 0.01, ***p < 0.001.

Similarly, NfL levels were significantly different between groups also after controlling for age, MMSE score and *APOE* genotype (F [2, 75] = 20.57, *p* < 0.001). Post hoc analysis showed that NfL concentration was higher in AD-d than in MCI AP- (*p* = 0.004, Cohen’s d = 1.223) and SCD AP− (*p* = 0.001, Cohen’s d = 1.167). No differences in plasma NfL levels were found among AD-d, MCI AP  and SCD AP+ . NfL concentration was higher in both MCI AP+ and SCD AP+ than in MCI AP− and in SCD AP− (MCI AP+ vs MCI AP− *p* = 0.001, Cohen’s d = 1.131; SCD AP+ vs MCI AP− *p* = 0.014, Cohen’s d = 1.257; MCI AP+ vs SCD AP− *p* < 0.001, Cohen’s d = 1.516; SCD AP+ vs SCD AP− *p* = 0.003, Cohen’s d = 1.700). No differences were found between SCD AP− and MCI AP− (*p* = 1.00, d = 0.272) (Table 2, Fig. 1).

### Accuracy of plasma p-tau181 and NfL in predicting AP status

We performed logistic regression analyses considering, in each analysis, A, T, N and AP status as dependent variables and plasma p-tau181 and NfL levels as covariates to obtain a combined model (NfL + p-tau181) in SCD and MCI, both taking separately and together (Supplementary materials). We did not consider AD-d patients since they were all AP+ . All the regression models were statistically significant and are described in Supplementary materials.

We performed ROC curve analyses to evaluate the diagnostic accuracy of plasma p-tau181, NfL and the NfL + p-tau181. AUCs for p-tau181, NfL and the combined model NfL + p-tau181 are reported in Table 3.

**Table 3:** Diagnostic accuracy of p-tau181, NfL and the combined model NfL + p-tau181 in predicting A, T, N and AP status.

		SCD	MCI	SCD + MCI
A	Log p-tau181	0.79 [0.61–0.96]	0.79 [0.61–0.96]	0.81 [0.71–0.92]
	Log NfL	0.80 [0.59–1]	0.80 [0.59–1]	0.77 [0.65–0.88]
	NfL + p-tau181	0.81 [0.62–1]	0.81 [0.62–1]	0.84 [0.75–0.94]
T	Log p-tau181	0.88 [0.75–1]	0.88 [0.75–1]	0.89 [0.81–0.96]
	Log NfL	0.88 [0.70–1]	0.88 [0.70–1]	0.82 [0.72–0.93]
	NfL + p-tau181	0.92 [0.81–1]	0.92 [0.81–1]	0.92 [0.86–0.98]
N	Log p-tau181	0.68 [0.48–0.89]	0.68 [0.48–0.89]	0.81 [0.71–0.91]
	Log NfL	0.69 [0.48–0.89]	0.69 [0.48–0.90]	0.74 [0.62–0.86]
	NfL + p-tau181	0.68 [0.48–0.89]	0.68 [0.48–0.89]	0.80 [0.70–0.90]
AP status	Log p-tau181	0.88 [0.68–1]	0.88 [0.76–1]	0.89 [0.81–0.96]
	Log NfL	0.88 [0.76–1]	0.88 [0.65–1]	0.81 [0.70–0.93]
	NfL + p-tau181	0.93 [0.80–1]	0.93 [0.80–1]	0.92 [0.86–0.99]

Values quoted in table are accuracy (in percentages %) and C.I., between brackets.

**Figure 2:**
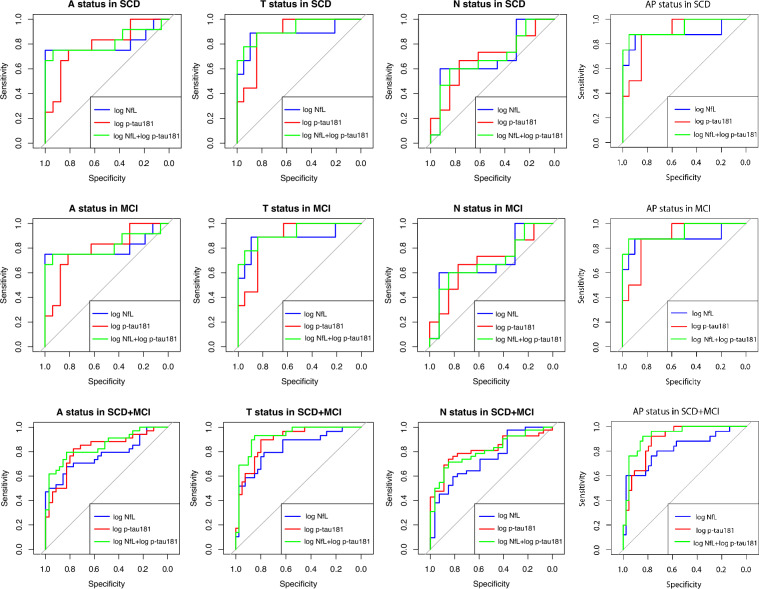
ROC curves for accuracy of plasma p-tau181, NfL and the combined model (NfL + p-tau181) in distinguishing A+ from A−, T+ from T−, N+ from N−, and AP+ from AP− patients in SCD and MCI, considering both separately and together.

Both plasma p-tau181 and NfL presented a good accuracy in discriminating A+ from A−, T+ and T−, N+ and N−, AP+ and AP− patients in SCD and MCI separately and the whole SCD and MCI group. The combined model did not significantly improve the accuracy of p-tau181 and NfL. However, despite not reaching the statistical significance, the combined model showed an excellent accuracy, with an AUC of 0.93 in SCD and MCI separately, and of 0.92 in the whole SCD + MCI group (Fig. 2).

### Concordance between plasma p-tau181 and NfL in SCD and MCI

Thirteen out of 43 SCD (30.23%) and 20 out of 41 MCI (48.78%) patients presented positive plasma p-tau181 (χ^2^ 3.02, *p* = 0.118, Cramer’s V 0.190). On the other hand, 6 out of 43 SCD (13.95%) and 11 out of 41 MCI (26.82%) patients presented positive NfL (χ^2^ 2.15, *p* = 0.179, Cramer’s V 0.160).

We analyzed concordance of plasma biomarkers in SCD and MCI patients. Plasma p-tau181 and NfL were concordant in 78.57% (95% C.I. 69.79–87.35) of cases: 50 out of 84 patients (59.52%) presented negative p-tau181 and NfL, while 16 patients (19.05%) showed both positive plasma biomarkers. Interestingly, plasma p-tau181 and NfL were discordant in 18 cases (21.43%), with just one patient (1.19%) with positive NfL and negative p-tau181, and 17 patients (20.24%) with positive p-tau181 and negative NfL (χ^2^ = 26.86, *p* < 0.001, Cramer’s V = 0.566) (Table 4).

**Table 4:** Concordance between plasma p-tau181 and in SCD and MCI.

		NfL		Total
		Negative	Positive	
p-tau181	Negative	50	1	51
	Positive	17	16	33
Total		67	17	84

Values quoted are frequencies of negative and positive patients for each biomarker.

Cohen’s K was significant (*p* < 0.001) with a value of 0.50, indicating a moderate concordance between plasma p-tau181 and NfL.

### Comparison of patients according to concordant or discordant plasma biomarkers

To explore the meaning of discordant results between NfL and p-tau181 with respect to A/T/N status, we considered only patients who underwent CSF analysis (69 patients). We divided these patients according to the concordance of plasma biomarkers into four subgroups: concordant negatives (NfL−/p-tau181−), concordant positives (NfL +/p-tau181 +), discordants with positive NfL (NfL +/p-tau181−), discordants with positive p-tau181 (NfL−/p-tau181 +).

- In concordant negative group (NfL−/p-tau181−), 35 out of 39 patients were AP− (true negative 89.74%) while 4 patients were AP+ (false negative 10.25%).
- In concordant positive group (NfL+ /p-tau181+), 14 out of 15 patients were AP+ (true positive 93.33%), while just one patient was AP− (false positive 6.66%).
- Only one patient was included in discordant NfL + /p-tau181− group. This patient was AP+ .
- In discordant NfL-/p-tau181 + group, 6 out of 14 (42.86%) patients were AP+ , while 8 (57.14%) patients were AP−.

### Effect of plasma biomarkers on the risk of presenting AD pathology

To estimate the risk of being AP+ based on positivity of p-tau181 and/or NfL in SCD and MCI patients, we performed a logistic regression analysis using p-tau and NfL as dichotomized (positive or negative) variables. The regression model was statistically significant (χ^2^ 37.45, *p* < 0.001). The model explained 57.4% (Nagelkerke R^2^) of the variance and correctly classified 84.10% of cases. Both the covariates (dichotomized plasma p-tau181 and NfL) had a statistically significant effect on the model (dichotomized p-tau181 B = 1.74, *p* = 0.002, OR =3.60, 95% C.I. 1.59–8.12; plasma NfL B = 0.141, *p* = 0.035, OR =1.51, 95% C.I. 1.01–1.31) (Table 5).

**Table 5:** Logistic regression model for AP status based of positivity of p-tau181 and/or NfL.

	**B**	***p***	**OR**	**95% C.I**	
				**Lower**	**Upper**
Plasma P-tau181	1.74	**0.015**	5.74	1.40	23.54
Plasma NfL	3.28	**0.004**	26.61	2.84	248.56

Regression coefficients (B), p-value (*p*), Odds Ratio (OR) and 95% Confidence Intervals (95% C.I.) for covariates included in the regression models are reported. Significant differences at *p* < 0.05, in bold characters.

Using the regression coefficients associated with the two covariates in the logistic model (p-tau181 and NfL), we defined the regression equation to estimate the risk of presenting an underlying AP status for each risk factor combination. The following equation describes the regression model:

$$logit\left( p \right) = \beta_{0} + \beta_{1} x_{1} + \beta_{2} x_{2} + \beta_{3} x_{3} + \cdots + \beta_{k} x_{k} {,}$$

$logit\left( p \right) = \ln \left( {\frac{p}{1 - p}} \right)$ is the logit function where p represents the probability that the event (i.e. “presenting an underlying AP status”) might happen, and β is the corresponding regression coefficient associated to each risk factor *x.* Entering the constant and the coefficients found in our logistic model, we obtained the following equation which enabled us to estimate the probability that the event “AP status” might happen. For each risk factor, value was “1” if the condition was satisfied (positive p-tau181 or positive NfL), “0” if the condition was not satisfied (negative p-tau181 or negative NfL).

$$\ln \left( {\frac{p}{1 - p}} \right) = - 2.10 + 1.74 \times^{\prime\prime}plasma p - tau181^{\prime\prime} + 3.28 \times^{\prime\prime}plasma NfL^{\prime\prime}$$

According to this model, risk of presenting an underlying AD pathology was:

- 10.91% (95% C.I. 3.55–18.27) if no risk factor was present,
- 41.10% (95% C.I. 29.49–52.71) and 76.49% (95% C.I. 66.48–86.50) if only one risk factor was present (respectively “positive p-tau181” and “positive NfL”),
- 94.88% (95% C.I. 89.69–100) if the two risk factors were both present (Table 6).

**Table 6:** Risk of presenting AP status for each risk factor combination.

Plasma p-tau181	Plasma NfL	Overall risk (%)	95% I.C	
			Lower	Upper
−	−	10.91	3.55	18.27
+	−	41.10	29.49	52.71
−	+	76.49	66.48	86.50
+	+	94.88	89.69	100

Overall risk was derived from the following regression model equation: logit p (presenting AD pathology) = −2.10 + 174 × (plasma p-tau181) + 3.28 × (plasma NfL). For each risk factor, value was “1” if the condition was satisfied (+), “0” if the condition was not satisfied (–).

## Discussion

There is an urgent need to move the use of plasma biomarkers from research settings to clinical practice, to define the correct application and to determine if the combined use might increase the accuracy for the early detection of AD. Our study fits into this scenario, trying to explore the combined use of plasma p-tau181 and NfL in SCD and MCI patients.

First, differences in both plasma p-tau181 and NfL levels among patients depended on the underlying pathology, not on diagnostic categories and the severity of cognitive decline. In more detail, plasma p-tau181 and NfL levels were similar in AD-d patients, MCI and SCD with underlying AD pathology. These finding suggested that differences between plasma biomarker levels in SCD, MCI, and AD-d patients were not driven by cognitive levels but rather by the underlying pathological substrate, as previously proposed by other studies^10–12,23–26^.

The accuracy of plasma p-tau 181 and NfL in detecting AD pathology were substantially similar in SCD and MCI patients. This might suggest that the accuracy of blood biomarkers is similar in both prodromal and preclinical AD, thus they might be promising tools in the very early stages of cognitive decline. Both plasma p-tau181 and NfL showed a good accuracy in detecting AP status in SCD and MCI both separately and together. Our results are in line with previous works showing a good accuracy of plasma p-tau181 in discriminating Aβ+ from Aβ− MCI and also Aβ+ from Aβ− cognitively unimpaired subjects^23^ and Aβ− healthy controls from Aβ+ “objectively defined” SCD^27^. However, the accuracy that we found was higher than those previously detected, probably because we did not consider only isolate Aβ positivity, but in combination with T biomarkers of A/T(N) system to define the presence of AD in our SCD and MCI patients.

Other studies had reported poorer accuracy of plasma NfL than those found in our work. Several works had demonstrated that plasma NfL had only fair accuracy in discriminating AD dementia from other neurodegenerative conditions, such as FTD^28–30^, and a moderate performance in discriminating AD-d patients and MCI due to AD from cognitively unimpaired subjects^30^. However, it has been reported that NfL accuracy is higher in SCD and in MCI than in patients with dementia^12^.

The combined model (NfL + p-tau181) allowed to obtain an excellent accuracy, reaching 0.92, even if this value was not significantly higher than accuracy of each single biomarker. This is not surprising, as plasma p-tau181 has already showed a high accuracy in detecting AD pathology^24^. On the other hand, the combined model NfL + p-tau181 was the only one presenting an AUC which exceeded 0.90. This might suggest that the combination of plasma biomarkers might give added value in predicting AD.

Despite other works proposed cut offs for plasma biomarkers, to the best of our knowledge, no previous studies tried to explore concordance and discordance of plasma biomarkers in predicting AD pathology. We investigated concordance between plasma p-tau181 and NfL in discriminating SCD and MCI patients carrying AD pathology from non-carriers according to cut offs previously defined by our group^11,12^ dichotomizing plasma biomarkers values in “positive” and “negative”. Interestingly, plasma p-tau181 and NfL showed only a moderate concordance. Therefore, we descriptively evaluated our cohort of patients who had undergone CSF analysis in order to shed light on the concordant and discordant cases, and thus on how to interpret the results of plasma biomarkers. First, the double positive concordance (both plasma p-tau181 and NfL positive) led to detection of AD pathology in 94% of cases. Nevertheless, the double negative concordance presented a risk of false negative of 10%, thus not allowing to completely exclude AD pathology. The most intriguing cases were those with discordant plasma biomarkers. Indeed, the only case with negative p-tau181 and positive NfL had a CSF AD profile. So, we can hypothesize that, despite negativity of plasma p-tau181, positive plasma NfL raise suspicion of an underlying neurodegenerative disease, warranting further investigation. On the other hand, discordant plasma biomarkers with positive p-tau181 but negative NfL identified AD pathology in 43% of SCD and MCI patients, but the remaining 57% were non-carriers of AD pathology. Consequently, isolated positivity of plasma p-tau181 was able to detect an underlying AD pathology in SCD and MCI patients, despite having a high risk of false positivity.

Considering the moderate concordance and the risk of false positives and negatives, these results support the idea that the combined use of plasma biomarkers may provide a better and more accurate detection of AD.

Finally, to further estimate the risk of presenting an AD pathology based on positivity of p-tau181 and/or NfL, we performed a logistic regression analysis using p-tau and NfL as dichotomized variables and we estimated the risk of presenting an underlying AD pathology for each risk factor combination. In line with the interpretation of concordant and discordant cases, we found that the highest risk of presenting AD pathology is present in case of the concurrent positivity of plasma p-tau181 and NfL (94%). The risk decreases to 76% in case of the isolated positivity of plasma NfL and to 43% in case of isolated p-tau181. These “discordant cases” are a challenging question and need to be further investigated via other biomarkers. First, the risk is significantly lower in case of isolated positive p-tau181 than in isolated NfL. Despite it has been widely demonstrated that plasma p-tau181 is a specific biomarker of the typical AD tauopathy, our results showed that its isolated positivity indicates a moderate risk of presenting an underlying AD pathology in MCI and SCD patients. These findings might be explained by the fact that our study is based on a relatively small cohort, and the cut offs proposed by our group need to be validated in further studies and in other populations; moreover, current research is trying to compare several isoforms of p-tau (p-tau217, p-tau181, and p-tau231) and several types of measures (mass spectroscopy assay vs Simoa immunoassay). It has been recently demonstrated that mass spectroscopy-based measures of p-tau217 showed the best performance and accuracy in discriminating Aβ+ from Aβ− MCI and progressors to dementia from non-progressors. Consequently, we might speculate that the risk of presenting an underlying AD pathology might be higher if another isoform of plasma p-tau (with higher specificity) were used^31^. The higher risk of AD pathology in case of isolated positivity of NfL compared to isolated positivity of p-tau181 might be due to two factors: first, the high sensitivity of NfL in detecting a neurodegenerative disease^28^, in particular in raising the suspicion of AD pathology in SCD and MCI patients^12^; second, the lower specificity of plasma p-tau181 as compared to other promising isoforms, such as p-tau217^32^.

Finally, the risk doesn’t reduce to zero, but remain at 10% even if both plasma biomarkers were negative. This suggests that patients who are ‘double positive’ may reliably exhibit an underlying AD pathology, although we cannot exclude the presence such pathology in those who are ‘double negative’. The inability to exclude AD pathology in case of double plasma biomarkers negativity is an intriguing and interesting finding. This may be linked to the risk of false negatives associated with the cut-off values proposed by our laboratory. Perhaps cut-off harmonization could reduce such risk. On the other hand, this data could be interpreted in the context of the selected patient population, since patients included in this study are not healthy controls but patients with a cognitive disorder, either objective or subjective.

**Figure 3:**
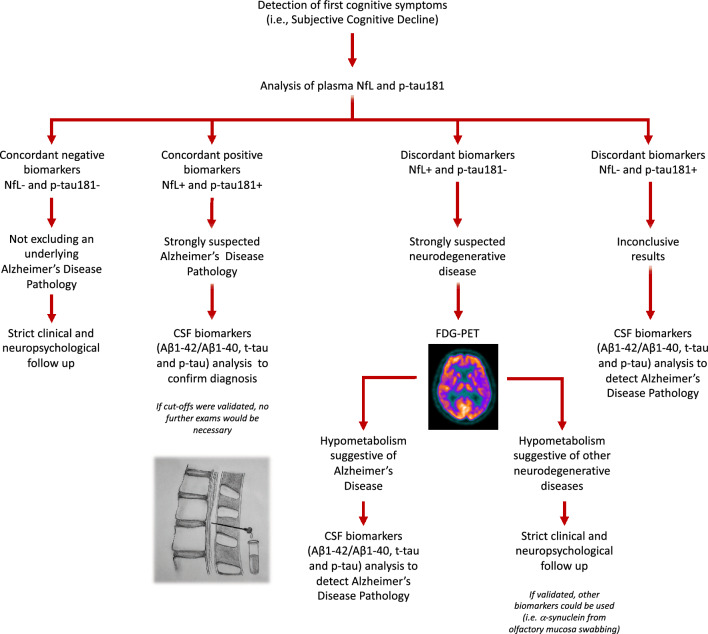
Flow chart for the potential use and interpretation of plasma biomarkers in clinical setting for the early detection of Alzheimer’s Disease.

Based on our results, we propose here a flow chart to guide the possible use of combined plasma biomarkers, in particular p-tau181 and NfL, in the clinical setting, considering patients in early stages of cognitive decline (i.e., SCD and MCI) (Fig. 3).

- In case both plasma p-tau181 and NfL are negative (concordant negative), an underlying AD pathology is not excludable. Therefore, close clinical and neuropsychological follow-up is recommended to assess any potential progression of the disturb.
- If both plasma p-tau181 and NfL are positive (concordant positive), it is highly suspicious that the cognitive impairment reported by the patient is due to AD. At the present time, patients would undergo more accurate investigations to confirm the diagnosis, in particular CSF Aβ1-42/Aβ1-40, t-tau and p-tau. If cut-offs were validated, and a high accuracy, sensitivity, and specificity of plasma biomarkers were established, we could hypothesize that CSF analysis might no longer be necessary.
- In cases of biomarker discordance, with positive NfL and negative p-tau181, a neurodegenerative disease is highly suspected, thus warranting further invasive investigations. In particular, we might suggest performing FDG-PET in order to identify potential hypometabolic patterns indicative of neurodegenerative disease^33^:
  - If FDG-PET were to show an hypometabolism suggestive (or at least partially indicative) of AD, CSF biomarker analysis (Aβ1-42/Aβ1-40, t-tau and p-tau) may be recommended.
  - If the FDG-PET were to indicate hypometabolism consistent with another neurodegenerative disease, in the future, if validated, other biomarkers could be used (i.e. α-synuclein from olfactory mucosa swabbing^34^); at present, we advise proceeding with clinical follow-up.
- In cases of discordance with negative NfL and positive p-tau181, the data are inconclusive, therefore, it is also advisable to continue the diagnostic process with additional investigations, particularly CSF biomarkers analisys (Aβ1-42/Aβ1-40, t-tau and p-tau), to confirm the suspicion of an underlying AD pathology due to plasma p-tau181 positivity or potentially rule out false positives.

Considering these premises, as plasma biomarkers continue to advance toward approval for clinical use, it is crucial to recognize the ethical implications that may arise. In a scenario where effective treatments are not yet available, the potential for diagnosing or strongly suspecting AD solely from blood analysis raises significant ethical considerations^35^. Such a diagnosis could have diverse consequences across different stages, raising questions about psychological well-being, personal autonomy, and societal implications. For instance, individuals with high plasma biomarker levels and only subtle cognitive disturbs may face uncertainties regarding activities such as driving or maintaining employment. Therefore, as plasma biomarkers transition into clinical practice, it becomes imperative to carefully consider the ethical dimensions and potential impact on patients’ lives.

Our work presents some limitations. First, the relatively small number of patients, which might reduce the power and generalizability of our study. Indeed, the second limitation is that, being a single-center study, there may be biases related to assessment and diagnosis procedures. Third, we did not include a sample of healthy control individuals. Fourth, the design of this study is cross-sectional: a longitudinal study should be performed in order to evaluate how plasma biomarkers levels change over time.

On the other hand, our study has some remarkable strengths. First of all, to the best of our knowledge, this is one of the first studies that tried to explore concordance and discordance of plasma biomarkers in detecting AD pathology in MCI and SCD. Secondly, patients were classified as carriers or non-carriers of Alzheimer’s pathology considering not only A status, but also the positivity of T and/or N biomarkers, while previous studies have considered the positivity of amyloid biomarkers alone. Our approach will increase the probability that patients with mild objective or Subjective Cognitive Decline are real carriers of Alzheimer’s pathology. Indeed, despite A+ /T−/N− patients are considered part of the Alzheimer’s continuum, they are properly classified as carriers of “Alzheimer’s pathological changes” and not Alzheimer’s Disease patients. Moreover, the presence of amyloid pathology alone in early stages of cognitive decline might not be specifically prognostic of conversion to dementia^36^. Third, this study clearly focused not only on MCI, but also on SCD, since it is an even earlier stage of cognitive decline, thus representing an intriguing target population to study and to identify those people at greatest risk of developing AD dementia. Finally, we suggested a flow chart for the potential use of plasma biomarkers in patients who concern with early, mild cognitive symptoms to illustrate the possible scenario for the clinical applicability an interpretation of plasma biomarkers.

In conclusion, our work perfectly fits in the current research landscape adopting a new paradigm that integrates peripheral biomarkers for a prompt diagnosis of AD focusing on pre-dementia stages, such as SCD^37^. Indeed, our work provides clinical insights into the use of plasma biomarkers for the early detection of AD, thus having potential implications for the clinical management of patients with SCD and MCI using these non-invasive tools. Moreover, we suggested that the combined use of plasma p-tau181 and NfL may give added value, thus providing more information for the correct interpretation and the detection of AD pathology. The combined use of plasma biomarkers may be potential applicable in clinical practice, particularly in SCD patients, leading to the identification of those individuals at greatest risk of developing AD dementia, which seem to be the ideal group in which to intervene with a specific treatment in order to stop neurodegeneration.

### Supplementary Information

Supplementary Table 1.

## Data Availability

All study data, including raw and analyzed data, and materials that support the findings of this study are available from the corresponding author (B.N.) upon reasonable request.
