# Supplementary material for: Future perspective and clinical applicability of the combined use of plasma phosphorylated tau 181 and neurofilament light chain in Subjective Cognitive Decline and Mild Cognitive Impairment
Source: Sci Rep. 2024 May 17;14:11307. doi: 10.1038/s41598-024-61655-6 (PMC11101654; doi:10.1038/s41598-024-61655-6)
Supplement: Supplementary file 1 — Supplementary Table 1. [file 41598_2024_61655_MOESM1_ESM.docx]

**Supplementary Materials**

**Table 1**

**Logistic Regression Model for A, T, N and AP status**

The regression model for A was statistically significant (χ^2^ 32.96, *p<*0.001). The model explained 47.5% (Nagelkerke R^2^) of the variance in conversion and correctly classified 77.30% of cases. Both the covariates (plasma p-tau181 and NfL) were statistically significant (plasma p-tau181 B=0.951, *p*=0.002, OR=2.59, 95% C.I. 1.41-4.72; plasma NfL B=0.134, *p*=0.027, OR=1.14, 95% C.I. 1.01-1.28).

The regression model for T was statistically significant too (χ^2^ 43.39, *p<*0.001). The model explained 62.8% (Nagelkerke R^2^) of the variance in conversion and correctly classified 84.10% of cases. Both the covariates (plasma p-tau181 and NfL) were statistically significant (plasma p-tau181 B=1.383, *p*=0.002, OR=3.98, 95% C.I. 1.68-9.43; plasma NfL B=0.163, *p*=0.030, OR=1.17, 95% C.I. 1.01-1.36).

The regression model for N was statistically significant (χ^2^ 26.99, *p<*0.001). The model explained 41.3% (Nagelkerke R^2^) of the variance in conversion and correctly classified 74.30% of cases. Among the covariates, only plasma p-tau181 was statistically significant (B=1.037, *p*=0.003, OR=2.82, 95% C.I. 1.42-5.57).

Finally, the regression model for AP status was statistically significant too (χ^2^ 40.99, *p<*0.001). The model explained 61.4% (Nagelkerke R^2^) of the variance in conversion and correctly classified 85.50% of cases. Both the covariates (plasma p-tau181 and NfL) were statistically significant (plasma p-tau181 B=1.281, *p*=0.002, OR=3.60, 95% C.I. 1.59-8.12; plasma NfL B=0.141, *p*=0.035, OR=1.51, 95% C.I. 1.01-1.31).

*Logistic Regression Model for A, T, N and AP status*

|  |  | **B** | ***p*** | **OR** | **95% C.I.** | |  |
| --- | --- | --- | --- | --- | --- | --- | --- |
|  |  |  |  |  | **lower** | **upper** | |
| A | Plasma p-tau181 | 0.951 | **0.02** | 2.59 | 1.41 | 4.72 |  |
|  | Plasma NfL | 0.134 | **0.027** | 1.14 | 1.01 | 1.28 |  |
| T | Plasma p-tau181 | 1.383 | **0.002** | 3.98 | 1.68 | 9.43 |  |
|  | Plasma NfL | 0.163 | **0.030** | 1.17 | 1.01 | 1.36 |  |
| N | Plasma p-tau181 | 1.037 | **0.003** | 2.82 | 1.42 | 5.57 |  |
|  | Plasma NfL | 1.01 | 0.104 | 1.10 | 0.98 | 1.25 |  |
| AP status | Plasma p-tau181 | 1.281 | **0.002** | 3.60 | 1.59 | 8.12 |  |
|  | Plasma NfL | 0.141 | **0.035** | 1.51 | 1.01 | 1.31 |  |

Regression Coefficients (B), p-value (*p*), Odds Ratio (OR) and 95% Confidence Intervals (95% C.I.) for covariates included in the regression models are reported. Significant differences at *p*<0.05, **in bold characters**.
